# Beyond information seeking: a latent profile analysis of financial media repertoires and psychological motivations among Chinese users

**DOI:** 10.3389/fpsyg.2026.1757025

**Published:** 2026-09-03

**Authors:** Qiren Xiang

**Affiliations:** School of Culture and Communication, Capital University of Economics and Business, Beijing, China

**Keywords:** financial media use, latent profile analysis, media repertoire, news avoidance, social media psychology, uses and gratifications

## Abstract

**Introduction:**

In the digital era, financial information acquisition is a complex behavioral outcome driven by individual psychological needs and structural constraints. Drawing on the Duality of Media framework and Uses and Gratifications (U&G) theory, this study investigates the cross-platform financial media repertoires of Chinese audiences and unpacks the psychological and structural mechanisms driving these configurations.

**Methods:**

Based on a survey of 1,002 participants, Latent Profile Analysis (LPA) was employed to identify distinct behavioral patterns. Multinomial logistic regressions, alongside subsample heterogeneity analyses, were conducted to examine how personal factors (including financial knowledge and psychological motivations) and structural factors predict repertoire membership.

**Results:**

The analysis identified five distinct repertoires: comprehensive Low-Frequency Repertoire, Comprehensive Medium-Frequency Repertoire, Social Media-Centric Repertoire, Comprehensive High-Frequency Repertoire, and Online-Only Repertoire. Repertoire configuration reflects a recursive interplay between individual agency and structural conditions. At the individual level, systematic financial knowledge alongside cognitive and belongingness motivations predicts high-engagement profiles, escapism suppresses active use, and affective motivation acts as a psychological buffer for the adoption of the Social Media-Centric Repertoire. Structurally, high-frequency omnivorous engagement hinges on time availability—supported by workplace hours and low-friction transit—while Online-Only Repertoire adoption is heavily concentrated within core metropolitan centers relative to lower-tier regions.

**Discussion:**

These findings expand Uses and Gratifications theory into a repertoire ecology framework, delineating spatiotemporal and generational boundary conditions. By illustrating how agency operates within structural bounds, this study provides targeted implications for professional media, digital platforms, and regulators to build an inclusive, balanced financial communication ecosystem.

## 1 Introduction

Against the backdrop of surging public demand for personal wealth management and the parallel expansion of the financial media sector, the importance of financial media has grown exponentially. This significance is particularly pronounced given that the general public often lacks the domain expertise required to accurately evaluate the economic implications and disruptive impacts of sudden crisis events, such as the COVID-19 pandemic (Haroon and Rizvi, 2020). Historically, mainstream media served as the primary source of financial information for the general public (Davis, 2006). However, the contemporary digital transition has fundamentally reconfigured this landscape. With the rapid proliferation of digital platforms—particularly social media, short-video feeds, and online financial communities—audience choices have expanded substantially (Wu, 2020). Today, users navigate a high-choice, multi-platform ecosystem where algorithmic recommendations and user-generated content (UGC) continuously compete with traditional news organizations.

Faced with an overwhelming array of media forms, one of the spontaneous coping mechanisms audiences adopt is establishing a “media repertoire.” A media repertoire does not imply exploring every available channel to its maximum; rather, it represents a specific, curated set of media selected from available options that align with the user's distinct needs and preferences (Hasebrink and Domeyer, 2012). To fully understand contemporary financial media consumption, scholarship must address two fundamental dimensions: what latent combinations of media users configure, and why such distinct configurations take shape.

However, a systematic review of the literature reveals two critical research gaps. First, in terms of analytical scope, most prior studies have focused in isolation on single platforms (such as social media alone or traditional news platforms alone), thereby failing to map the holistic, cross-platform media repertoire curated by real-world audiences. Second, and more importantly, existing repertoire-related scholarship has largely centered on broader constructs, such as channel repertoires (Heeter, 1985), information repertoires (Reagan, 1996), news repertoires (Molyneux, 2019), or social media repertoires (Dvir-Gvirsman, 2022). Financial media consumption, characterized by greater cognitive and emotional complexity, remains an unexplored domain for repertoire analysis.

To bridge these gaps, this study adopts the media repertoire framework to map the latent typologies of financial media consumption (RQ1), while integrating the Uses and Gratifications (U&G) paradigm and the Duality of Media framework to examine how individual psychological motivations and structural factors co-construct these distinct configurations (RQ2). Accordingly, two overarching Research Questions are proposed:

RQ1: What distinct financial media repertoires exist among Chinese users when evaluated through a person-centered, multi-platform lens?

RQ2: How do psychological motivations and structural factors interact to predict membership in these financial media repertoires?

Theoretically, by introducing the concept of the “financial media repertoire,” this work not only extends the repertoire framework into a specialized, high-risk domain but also enriches the Uses and Gratifications (U&G) paradigm by revealing how psychological motivations shape cross-platform financial media choices in digital environments. Practically, by uncovering the heterogeneous nature of financial media consumption, this study offers a pragmatic foundation for financial regulators, mainstream news outlets, and digital platforms. It delivers a strategic framework to move beyond “one-size-fits-all” communication, enabling stakeholders to design segmented, audience-tailored information strategies and behavioral interventions in high-choice digital ecosystems.

## 2 Literature review and theoretical framework

### 2.1 The media repertoire framework

In contemporary media-rich environments, the simultaneous adoption of multiple media platforms has become a fundamental feature of audience behavior. Within multi-screen environments, media usage is no longer a “zero-sum” game; rather, it is characterized by complementary and additive dynamics, where audiences strategically utilize different screens to fulfill distinct personal needs based on technological affordances, ease of use, content availability, and individual lifestyles (Phalen and Ducey, 2012). Recognizing this shift, communication scholars have increasingly advocated for a “repertoire-oriented” approach (Hasebrink and Domeyer, 2012; Hasebrink and Popp, 2006). Measuring the consumption of a single isolated medium provides only a partial picture of audience behavior. Instead, analyzing a “media repertoire”—defined as the stable, curated set of media channels routinely utilized by an individual—offers a holistic, audience-centric framework to examine cross-platform media consumption.

The empirical and conceptual foundations of the repertoire framework were initially laid during the cable television era under the construct of “channel repertoires,” defined by Heeter (1985) as the distinct subset of channels regularly viewed by an individual or household from an expanding array of options. Early research focused heavily on identifying the structural and demographic predictors of repertoire size. Structural constraints, particularly cable subscription status and audience availability (operationalized as time allocated to viewing), emerged as dominant explanatory variables; for instance, Yuan and Webster (2006) demonstrated that cable subscription and time spent viewing accounted for 65% of the variance in channel repertoire size. While demographic variables generally exerted limited influence, some scholars observed that age and education significantly predicted self-reported repertoires (Ferguson and Perse, 1993; Neuendorf et al., 2001). However, scholarship during this era remained predominantly confined to evaluating repertoire size and channel volume, paying scant attention to the internal qualitative composition and multi-platform synergies of these channel configurations.

To transcend the confines of single-medium television studies, Reagan (1996) introduced the information repertoire to describe the curated set of information sources individuals routinely access to acquire specific types of information. This audience-centric shift enabled researchers to investigate domain-specific information-seeking behaviors, such as how populations curate distinct media sets to obtain preventive health care information (O'Keefe et al., 1998). Building upon this foundation, “news repertoires” emerged to capture how users combine various news platforms—offline press, broadcasting, and digital outlets—into comprehensive, cross-media news consumption patterns, shifting analytical focus away from platform-level market competition toward holistic user diets (Molyneux, 2019).

With the rapid expansion of mobile internet and algorithmic platforms, contemporary empirical scholarship has increasingly utilized person-centered typological approaches to identify distinct media and news repertoires. Taneja et al. (2012) mapped four distinct media repertoires closely tied to daily routine and audience availability: “home media” (TV-dominant), “work media” (PC-dominant), “non-work online media,” and “mobile media.” In the mobile news era, Wolf and Schnauber (2015) identified six distinct news repertoires, ranging from traditional-heavy diets to mobile-only and digital-centric patterns. More recently, empirical studies in algorithmically curated social media environments have consistently documented a stratification of news consumption. Scholars have identified recurring typologies across diverse cultural contexts, including “news avoiders” (characterized by low cross-platform engagement), “digital/mobile-centric users,” “traditional-social hybrids,” and “comprehensive news seekers” (Dvir-Gvirsman, 2022; Kim and Schwarze, 2021; Palmer and Toff, 2020; Peters et al., 2022; Toff and Kalogeropoulos, 2020). A prevailing pattern across recent youth and digital repertoire studies is the systematic retreat from traditional press and the ascendancy of algorithm-driven digital platforms (Peters et al., 2022).

Fundamentally, a repertoire represents an individualized, self-regulatory strategy developed by audiences to manage information overload in an age of rapid content proliferation. Tracing the conceptual evolution from channel repertoires to information and news repertoires demonstrates that the repertoire framework serves as an enduring lens for examining selective exposure and active audience agency. However, existing scholarship has largely applied this framework to general news or broad information-seeking contexts. Specialized media domains—most notably financial media consumption—involve higher cognitive complexity, direct economic risk, and intense emotional volatility, remaining an unmapped territory for repertoire analysis.

To bridge this theoretical gap, this study conceptualizes the “financial media repertoire”—defined as the stable, cross-platform configuration of traditional, digital, social, and specialized financial media channels routinely utilized by an individual for financial information processing. By extending the repertoire framework into the specialized domain of financial communication, this study provides a granular, person-centered mapping of how modern users curate cross-platform financial diets. Accordingly, the first research question is derived:

RQ1 (What): What distinct financial media repertoires exist among Chinese users when evaluated through a person-centered, multi-platform lens?

### 2.2 Uses and gratifications paradigm

The Uses and Gratifications (U&G) paradigm represents one of the most enduring audience-centric theoretical frameworks in communication scholarship (Bryant and Miron, 2004). The conceptual genesis of U&G emerged during a pivotal moment of disciplinary reflection, when Berelson (1959) famously declared that communication research was dying. Responding to this pessimism, Katz (1959) argued that what was dissipating was merely the persuasion-centric model of mass communication, whereas an expansive domain remained if scholarship shifted its core inquiry from “what media do to people” to “what people do with media.” Early foundational efforts cataloged audience motives into functional dimensions, culminating in McQuail (1972) fourfold typology—diversion, personal relationships, personal identity, and surveillance—and the formalization of the U&G model by Katz et al. (1973), Katz et al. (1974). By placing self-aware, goal-directed individuals at the analytical center, the paradigm established that media selection is propelled by social and psychological needs that generate specific expectations of media sources, ultimately leading to differential patterns of exposure and need gratification.

As the paradigm evolved across traditional broadcasting environments, scholars systematically cataloged and categorized the empirical dimensions of media motivations. Early empirical investigations identified a wide array of psychological drivers underlying media use, including learning, habit, relaxation, companionship, stimulation, and escapism from real-world difficulties (Rubin, 1977, 1983). Across these diverse cataloging efforts, U&G scholarship increasingly demonstrated that audience members selectively engage with specific media channels based on their expected utility. Rather than viewing audience motivations as erratic or random, researchers established that media consumption is governed by structured, goal-directed psychological orientations, laying the foundation for understanding how individuals actively curate their media menus to satisfy multi-faceted psychological needs, particularly in utilizing surveillance gratifications to acquire domain-specific knowledge (Vincent and Basil, 1997).

In contemporary digital and networked environments, the U&G paradigm has demonstrated exceptional vitality in explaining user adoption and platform engagement (Stafford and Gonier, 2004). While early studies revealed weak correlations between legacy media motives and online adoption (Lin, 1999), subsequent scholarship updated the paradigm to account for interactive affordances. Stafford and Stafford (2001) expanded gratification taxonomies into three overarching categories: content gratifications (retrieving specific information), process gratifications (the functional, habitual experience of navigating the medium itself), and social gratifications (interpersonal connection and networking). In recent years, empirical research across emerging digital domains—such as messaging apps, live-streaming e-commerce, augmented reality, and over-the-top (OTT) streaming (Gan and Li, 2018; Bossen and Kottasz, 2020; Ibáñez-Sánchez et al., 2022; Menon, 2022)—has repeatedly affirmed that psychological gratifications cluster around cognitive/utilitarian needs, affective/hedonic fulfillment, and social connectedness.

In summary, the diverse array of media motivations documented across the empirical U&G literature aligns seamlessly with the classic typology synthesized by West et al. (2010), which categorizes audience gratifications into fundamental dimensions such as cognitive, affective, personal integrative, social integrative, and tension-release (escapism) needs. Grounded in this established theoretical synthesis, the present study primarily draws upon these core psychological motivations to analyze audience agency within the high-stakes context of financial media consumption. Together, these dimensions offer a comprehensive theoretical framework for explaining how psychological drivers shape individual media choices.

### 2.3 The duality of media framework

Research on audience media choice has traditionally developed along two distinct analytical trajectories. The first trajectory privileges individual-centric explanations, asserting that media selection is primarily driven by audience preferences, psychological states, and subjective needs. Theoretical frameworks such as the Uses and Gratifications paradigm (Katz et al., 1974) and mood management theory (Zillmann, 1988) exemplify this perspective by positioning individual predispositions as the chief determinants of media exposure. Conversely, the second trajectory emphasizes the overarching influence of structural factors, arguing that media accessibility, technological affordances, and macro-environmental constraints govern audience behavior (Webster, 2009). These two perspectives resonate strongly with Webster (2011) framework of the “Duality of Media,” which adapts Giddens (1984) structuration theory and the concept of the “Duality of Structure.” The duality framework posits a recursive relationship in which structure and agency are mutually constitutive: structural forces establish the boundaries and affordances within which individuals operate, while active agents utilize available structural resources to reproduce social and media systems. In the context of media repertoires, users leverage ambient structural resources to fulfill personal motives, yet their choices remain bounded by structural conditions such as device availability, regulatory environments, and temporal constraints.

Within this dual framework, individual-level attributes and personal capabilities serve as fundamental drivers of repertoire configuration (Kim and Viswanathan, 2015; Wonneberger et al., 2011). Extensive empirical scholarship demonstrates that demographic characteristics—specifically age, education, and socio-economic status—significantly condition repertoire diversity and composition (Edgerly, 2015; Kim, 2016). Age operates as a vital predictor of digital vs. traditional orientation; younger demographics tend to curate multi-platform, digital-first news repertoires, whereas older cohorts rely predominantly on legacy broadcasting and print press (Edgerly, 2015; Kim, 2016). Furthermore, age influences repertoire breadth, with younger users consuming news across a wider variety of digital channels compared to the narrower, single-medium diets of older users (Diehl et al., 2019). Socioeconomic status and education level further moderate repertoire scope. Higher educational attainment correlates with more diversified, cross-platform media diets across broadcast, digital, and print outlets, whereas lower education levels tend to constrain consumption toward dominant, highly accessible channels like television (Sumida et al., 2019).

Simultaneously, structural factors define the spatial and temporal environment within which repertoires take shape. Regional geography constitutes a critical structural condition, as media infrastructure and channel availability vary substantially across physical locations. Individuals residing in metropolitan areas benefit from dense media environments and advanced digital infrastructure, facilitating active adoption of mobile news portals and niche outlets, whereas rural populations face structural limitations that favor legacy media reliance (Kim, 2016). Beyond spatial infrastructure, temporal structure is governed by “audience availability”—the structural time budget determined by an individual's daily routines, work commitments, and lifestyle demands (Yuan, 2011; Yuan and Webster, 2006). Audience availability dictates when and for how long media can be accessed, operating as a potent structural constraint that accounts for substantial variance in repertoire size and composition (Yuan and Webster, 2006; Wonneberger et al., 2011).

In summary, the formation of specialized financial media repertoires cannot be fully understood through isolated psychological motivations or structural constraints alone. Rather, repertoire membership emerges from the recursive interplay between individual agency—driven by psychological motivations and personal capabilities such as educational attainment—and structural conditions, such as regional geography and audience availability. To empirically investigate how these dual mechanisms co-construct specialized cross-platform consumption patterns, the second research question is derived:

RQ2 (Why): How do psychological motivations and structural factors interact to predict membership in these financial media repertoires?

## 3 Method

### 3.1 Sampling and data collection

Data collection was conducted via a leading professional third-party online research platform (Tencent Cloud) in China. The utilization of online survey panels for empirical data collection has become increasingly prevalent and widely accepted across social science disciplines (Kees et al., 2017). Regarding the sampling strategy, this study adopted a quota sampling approach—an established methodological precedent in cross-platform media repertoire scholarship (Kim, 2016). Given the absence of official census data specifically tracking “financial media users,” this study utilized the latest *Survey Report on Mutual Fund Investors in China* released by the Asset Management Association of China (AMAC) to set the demographic characteristics of the sample.

Methodologically, it is crucial to emphasize that the primary objective of this study lies in exploratory pattern identification and relational impact analysis, rather than conducting descriptive population parameter inferences. When research aims at theory application and structural relation testing rather than population parameter estimation, strict population-level representativeness is not an essential prerequisite—rather, non-probability panel sampling with domain-aligned quota controls provides a valid basis for evaluating theoretical mechanisms, provided that the sample captures sufficient target heterogeneity (Calder et al., 1981).

Furthermore, empirical research confirms that while convenience or online panel samples may exhibit minor main-effect baseline differences, they do not produce significant interaction or relational differences compared to general adult populations (Snook, 2011). In empirical communication and behavioral scholarship, recruiting participants through non-probability online surveys—even via straightforward web distributions without formal quota constraints—represents an established precedent for measuring media usage and psychological outcomes (Wang et al., 2017).

A total of 1,826 questionnaires were distributed across different geographical regions in China. After applying stringent data-cleaning filters to eliminate incomplete responses, straight-lining, failed attention checks, and unrealistically short completion times, 1,002 valid questionnaires were retained for analysis, yielding an effective response rate of 54.87%. This response rate comfortably satisfies and exceeds widely recognized methodological benchmarks for individual-level online surveys (Baruch and Holtom, 2008). For the primary analytical technique—Latent Profile Analysis (LPA)—*N* = 1,002 also significantly surpasses the standard threshold, thereby ensuring high statistical power (Nylund et al., 2007).

This study did not involve any experimental interventions. Informed consent was obtained digitally from all participants prior to the survey; they were informed that participation was voluntary, anonymous, and that data would be used strictly for academic purposes.

### 3.2 Measures

Measurement scales were adapted from established literature. Prior to formal survey administration, preliminary qualitative interviews were conducted with financial media users to refine item phrasing, ensure contextual readability, and validate the media channel taxonomy within the Chinese context.

#### 3.2.1 Financial media usage

Drawing upon foundational domestic scholarship in Chinese financial communication (Wu, 2020) and qualitative insights from preliminary user interviews, this study categorized the financial media landscape based on transmission channels. The financial media ecosystem was classified into six broad domains and further subdivided into nine granular channel types across offline and online spheres: (1) offline channels, comprising financial journals, financial newspapers, and financial broadcast and TV (3 types); (2) online information platforms, subdivided into mobile-based apps/sites and PC-based websites (2 types); (3) financial communities, subdivided into forum-based communities and instant messaging (IM)-based communities (2 types); and (4) financial influencers, subdivided into video-based financial influencers and non-video-based financial influencers (2 types).

Operationally, following established methodological precedents in media usage and repertoire scholarship (Andersen et al., 2022; Edgerly, 2015), consumption frequency for each of the nine media channels was measured using single-item indicators. Participants rated their media usage on a 7-point ordinal scale coded from 1 to 7: 1 = Never, 2 = 1–3 times a year, 3 = 1–3 times every 6 months, 4 = 1–3 times a month, 5 = 1–3 times a week, 6 = 4–7 times a week, and 7 = Multiple times daily.

#### 3.2.2 Financial media use motivations

Grounded in the Uses and Gratifications (U&G) framework (West et al., 2010), audience motivations for financial media consumption were operationalized across five functional need categories comprising seven distinct dimensions. All items were scored on a 7-point Likert scale (1 = Strongly Disagree to 7 = Strongly Agree). Informed by preliminary qualitative interviews, items were contextualized for Chinese financial consumers—specifically expanding cognitive motivation to capture reliance on peer opinions and actions alongside objective financial news. To maintain a strictly consistent reporting standard across all constructs, the item count, internal consistency, functional definition, sample item, and scale source for each motivation dimension are detailed in Table 1.

**Table 1:** Measurement items, functional definitions, and sources for financial media use motivations.

Motivation	Items	Source
Affective	1. I use financial media because it can be entertaining.	Sjöblom and Hamari (2017)
	2. I use financial media because it is appealing.	
	3. I use financial media to make myself happy.	
Cognitive	1. I use financial media because the information provided is useful.	Lim (2009)
	2. I use financial media to learn more financial information.	
	3. I use financial media to acquire financial knowledge I did not know before.	
	4. I use financial media to obtain information that helps me make financial decisions.	
	5. I use financial media to learn more about others' opinions.	
	6. I use financial media to observe how others are acting/investing.	
Personal integration	1. I use financial media so that my opinions on finance are considered by others.	Hernandez et al. (2011)
	2. I use financial media so that my opinions are considered professional by others.	
	3. I use financial media to improve my reputation among those around me.	
Companionship	1. I use financial media to avoid feeling lonely.	(Smock et al. 2011)
	2. I use financial media when there is no one else to talk to or be with.	
	3. I use financial media to reduce my sense of loneliness.	
Belongingness	1. I use financial media to maintain relationships with others.	Hernandez et al. (2011)
	2. I use financial media to find partners/peers who share my interests.	
	3. I use financial media to feel like I belong to a certain group.	
Escapism	1. I use financial media to forget about school, work, or other things. 2. I use financial media to get away from family or others for a while. 3. I use financial media to put off something I should be doing.	Smock et al. (2011)
Habitual use	1. I use financial media just because I like to browse the content casually.	Smock et al. (2011)
	2. I use financial media out of habit; it is just something I do.	
	3. I use financial media when I have nothing better to do.	
	4. I use financial media to pass time, especially when I am bored.	
	5. I use financial media to relax from stress/tension.	
	6. I use financial media to take a break.	

To evaluate the construct validity and psychometric properties of the seven-factor financial media motivation scale, a Confirmatory Factor Analysis (CFA) was conducted using Mplus 8.0 with the Robust Maximum Likelihood (MLR) estimator. The empirical data demonstrated an excellent overall fit to the hypothesized seven-factor measurement model: χ^2^(303)= 1,483.904, *p* < 0.001, χ^2^/*df* = 4.898; Comparative Fit Index (CFI) = 0.934; Tucker-Lewis Index (TLI) = 0.924; Root Mean Square Error of Approximation (RMSEA) = 0.062 [90% CI (0.059, 0.066)]; and Standardized Root Mean Square Residual (SRMR)= 0.057. As shown in Table 2, all 27 observed items loaded significantly on their respective latent motivation dimensions (*p* < 0.001). Standardized factor loadings ranged from 0.681 to 0.944, with 26 out of 27 items exceeding the stringent threshold of 0.80. The *R*^2^ values for observed items ranged between 0.464 and 0.892, confirming strong indicator reliability and robust convergent validity across all seven motivation constructs.

**Table 2:** Measurement model, factor loadings, reliability, and convergent validity.

Dimension/construct	Item	Standardized factor loading (λ)	Cronbach's α	Composite Reliability (CR)	Average variance extracted (AVE)
Affective motivation	M1	0.894	0.913	0.914	0.779
	M2	0.901			
	M3	0.852			
Cognitive motivation	M4	0.896	0.953	0.954	0.774
	M5	0.920			
	M6	0.905			
	M7	0.836			
	M8	0.856			
	M9	0.864			
Personal integration	M10	0.803	0.900	0.901	0.753
	M11	0.899			
	M12	0.897			
Social integration-companionship	M13	0.911	0.917	0.921	0.796
	M14	0.830			
	M15	0.933			
Social integration-belongingness	M16	0.912	0.925	0.924	0.802
	M17	0.895			
	M18	0.879			
Tension release-escapism	M19	0.891	0.943	0.945	0.851
	M20	0.944			
	M21	0.932			
Tension release-habitual use & relaxation	M22	0.681	0.933	0.933	0.702
	M23	0.774			
	M24	0.851			
	M25	0.893			
	M26	0.914			
	M27	0.891			

All factor loadings are significant at *p* < 0.001. Recommended academic cut-offs: Cronbach's α > 0.70, CR > 0.70, and AVE > 0.50.

Discriminant validity was evaluated using the Fornell and Larcker (1981) criterion. As presented in Table 3, the square roots of the AVE for all seven constructs ranged from 0.838 to 0.922, consistently exceeding their inter-construct correlations across nearly all pairings. To rigorously address the high correlation between Companionship and Belongingness (*r* = 0.927), a nested model comparison was conducted. Merging these two dimensions into a single “Social Integration” factor led to a statistically significant degradation in model fit compared to the baseline seven-factor model (Δχ^2^(6) = 104.80, *p* < 0.001; ΔCFI = 0.007; ΔAIC = 219.70). These findings confirm that all seven motivation dimensions represent empirically distinct constructs.

**Table 3:** Discriminant validity assessment.

Construct	AFF	COG	PER	COM	BEL	ESC	HAB
**AFF**	**0.883**						
**COG**	0.761	**0.880**					
**PER**	0.786	0.625	**0.868**				
**COM**	0.731	0.453	0.876	**0.892**			
**BEL**	0.757	0.562	0.867	0.927	**0.896**		
**ESC**	0.590	0.331	0.786	0.868	0.829	**0.922**	
**HAB**	0.717	0.527	0.753	0.821	0.826	0.819	**0.838**

Bold diagonal elements represent the square root of the Average Variance Extracted for each construct. Off-diagonal elements are the inter-construct correlation coefficients (r) derived from the STDYX output in Mplus. All correlations are significant at *p* < 0.001.

To evaluate potential Common Method Bias (CMB) across the seven psychological motivation constructs (measured by 27 items), an Unmeasured Latent Method Factor (ULMF) approach with equal method loading constraints was performed in Mplus 8.0 (Podsakoff et al., 2003). Specifically, a global latent method factor was specified to load on all 27 motivation items with constrained equal unstandardized loadings, while constraining its covariances with all seven substantive motivation factors to zero.

The ULMF model demonstrated satisfactory overall goodness-of-fit: χ^2^ = 1725.640, df = 302, *p* < 0.001; CFI = 0.954; TLI = 0.947; RMSEA = 0.069 [90% CI (0.065, 0.072)]. Crucially, after controlling for the latent method factor, the substantive factor loadings for the motivation constructs remained statistically significant (*p* < 0.001), with standardized loadings ranging from 0.413 to 0.753. These results confirm that while a common method artifact exists, the substantive motivation constructs retain robust construct validity, demonstrating that common method variance does not artifactually distort the measurement model.

Given the high factor loadings (λ > 0.70) and strong internal consistency established in the CFA, the items for each motivational dimension were averaged to create composite scores for subsequent analyses.

#### 3.2.3 Structural factors and demographics

Following Taneja et al. (2012), structural audience availability was operationalized as time constraints shaping individual media consumption opportunities, measured via three ordinal temporal indicators: (1) daily work or study hours, (2) awake time spent at home, and (3) daily commute duration. Demographic and socio-economic variables in this study encompass gender, age, personal and household annual income, education level, occupation, systematic financial learning experience, and geographic residence.

Regarding variable coding, binary dummy variables were created for gender (0 = female, 1 = male), systematic financial learning (0 = no, 1 = yes), occupation (0 = non-financial, 1 = financial sector), and geographic residence (1 = yes, 0 = no for each of the five administrative tiers). Ordinal variables—including personal and household annual income (1–7), education level (1–5), daily work/study hours (1–4), awake time at residence (1–4), and daily commute duration (1–4)—were assigned numeric values in ascending order of their respective interval categories, while age was measured as a continuous variable. The detailed survey items, response options, and sample frequency distributions for all structural factors and demographic variables are presented in Table 4.

**Table 4:** Survey questionnaire design, items, response options, and sample frequency.

Variable	Survey item	Response options	Frequency
Age	What is your age?	Continuous (open-ended)	Mean = 36.63
Gender	What is your gender?	Male	548
		Female	454
Personal annual income	What is your personal annual income level?	Less than 50,000 RMB	374
		50,000–100,000 RMB	347
		100,001–200,000 RMB	180
		200,001–300,000 RMB	58
		300,001–600,000 RMB	28
		600,001–1,000,000 RMB	8
		More than 1,000,000 RMB	7
Household annual income	What is your total household annual income level?	Less than 50,000 RMB	148
		50,000–100,000 RMB	235
		100,001–200,000 RMB	341
		200,001–300,000 RMB	131
		300,001–600,000 RMB	107
		600,001–1,000,000 RMB	24
		More than 1,000,000 RMB	16
Education level	What is your highest completed level of education?	Middle school or below	63
		High school/secondary vocational/technical school	178
		Associate degree (Junior college)	265
		Bachelor's degree	424
		Master's degree or above	72
Systematic financial learning	Have you systematically learned financial knowledge?	Yes	284
		No	718
Occupation	What is your occupation?	Finance-related practitioner/student	119
		Non-finance practitioner/student	883
Daily work/study hours	How much time do you spend on work or study per day?	Less than 4 h	345
		4–8 h	341
		8–12 h	291
		More than 12 h	25
Awake time at residence	Excluding sleep, how much awake time do you spend at your residence per day?	Less than 4 h	373
		4–8 h	355
		8–12 h	175
		More than 12 h	99
Daily commute time	How long is your daily commute time?	Less than 30 min	458
		30–60 min	337
		60–90 min	97
		More than 90 mins	110
Geographic residence	Which region/administrative tier do you currently reside in?	Municipality	205
		Provincial capita	246
		Prefecture-level city	240
		County-level city/county	192
		Township/Town/Village	119

For subsample heterogeneity analyses, geographic residence was dichotomized into Core Urban (municipalities and provincial capitals, *n* = 451) and Non-Core Urban (prefecture-level cities, county-level cities, and rural areas, *n* = 551).

### 3.3 Statistical analysis

Statistical analyses were performed using IBM SPSS Statistics 26.0 and Mplus 8.0, with specific tasks allocated to each software platform. IBM SPSS Statistics 26.0 was used for data cleaning, preprocessing, scale reliability testing (Cronbach's α), computing descriptive statistics, and executing multinomial and binary logistic regression analyses. Mplus 8.0 was employed for construct validity testing (Confirmatory Factor Analysis) and conducting Latent Profile Analysis (LPA) to classify respondents into distinct subgroups based on financial media usage patterns.

## 4 Results

### 4.1 Descriptive statistics and bivariate correlations

As presented in Table 5, continuous composite mean scores were calculated for the seven financial media use motivation dimensions on a 7-point Likert scale. Among the primary motivations, Cognitive Motivation exhibited the highest mean score (*M* = 4.85, SD = 1.59), indicating that information acquisition and surveillance remain the predominant drivers for financial media consumption. This was followed by Affective Motivation (*M* = 4.08, SD = 1.64) and Personal Integration (*M* = 3.70, SD = 1.71). In contrast, tension-release and social integration dimensions yielded relatively lower averages, with escapism receiving the lowest mean score (*M* = 2.92, SD = 1.77).

**Table 5:** Descriptive statistics and bivariate correlations.

Variable	*M*	SD	1	2	3	4	5	6	7
Affective	4.08	1.64	—						
Cognitive	4.85	1.59	0.707^***^	—					
Personal integration	3.70	1.71	0.731^***^	0.618^***^	—				
Companionship	3.25	1.74	0.704^***^	0.464^***^	0.803^***^	—			
Belongingness	3.49	1.76	0.706^***^	0.546^***^	0.786^***^	0.861^***^	—		
Escapism	2.92	1.77	0.572^***^	0.336^***^	0.720^***^	0.816^***^	0.781^***^	—	
Habit & relaxation	3.66	1.63	0.684^***^	0.552^***^	0.695^***^	0.763^***^	0.764^***^	0.770^***^	—

*M* = Mean; SD =Standard Deviation. All bivariate correlations are two-tailed. ^***^ *p* < 0.001.

Bivariate correlation analyses revealed statistically significant positive correlations among all seven motivation dimensions (*p* < 0.001). Cognitive motivation showed moderate-to-strong positive relationships with affective motivation (*r* = 0.71, *p* < 0.001) and personal integration (*r* = 0.62, *p* < 0.001), but a weaker association with escapism (*r* = 0.34, *p* < 0.001). Meanwhile, sub-dimensions under the same broader functional umbrella—such as companionship and belongingness (*r* = 0.86, *p* < 0.001)—demonstrated strong linear associations, consistent with the measurement model specification.

### 4.2 Typology of users' financial media repertoires

To identify distinct patterns in financial media usage, Latent Profile Analysis (LPA) was conducted using Mplus 8.0. The indicators comprised the usage frequency across the nine types of financial media. Since usage frequency was measured on an ordinal scale, the values were treated as continuous variables for the purpose of profile estimation, a common practice in repertoire studies.

Model fit was evaluated using a set of indices recommended by Nylund-Gibson et al. (2019), including the Akaike Information Criterion (AIC), Bayesian Information Criterion (BIC), sample-size adjusted BIC (aBIC), Entropy, the Lo–Mendell–Rubin likelihood ratio test (LMR-LRT), and the bootstrap likelihood ratio test (BLRT). Lower values for AIC, BIC, and aBIC indicate a better fit. Significant *p*-values (*p* < 0.05) for LMR-LRT and BLRT suggest that the k-class model offers a statistically significant improvement over the (*k*−1)-class model. Higher entropy values (ranging from 0 to 1) reflect greater classification precision.

As presented in Table 6, the fit indices (AIC, BIC, aBIC) continued to decrease as the number of classes increased. However, the LMR-LRT *p*-value for the 6-class model was non-significant (*p* = 0.486), indicating that adding a sixth class did not provide a statistically significant improvement over the 5-class solution. Considering the fit statistics alongside the theoretical interpretability and distinctiveness of the profiles, the 5-class model was selected as the optimal solution.

**Table 6:** Goodness-of-fit indices for latent profile models.

Model	AIC	BIC	aBIC	pLMR	pBLRT	Entropy	Sample Size (*n*)
1	37,885.977	37,974.352	37,917.183	-	-	-	1002
2	33,998.628	34,136.101	34,047.171	0.0000	0.0000	0.927	632/370
3	32,852.281	33,038.851	32,918.161	0.0009	0.0000	0.893	407/334/261
4	32,293.203	32,528.871	32,376.421	0.0096	0.0000	0.901	384/148/146/324
5	31,828.654	32,113.420	31,929.208	0.0303	0.0000	0.906	386/217/152/118/129
6	31,519.058	31,852.922	31,636.949	0.4859	0.0000	0.907	144/170/127/370/105/86

Table 7 illustrates the estimated mean usage frequencies for the five identified profiles:

**Table 7:** Results of latent profile analysis for financial media repertoires.

Financial media type	Profile 1: comprehensive low-frequency repertoire	Profile 2: comprehensive medium-frequency repertoire	Profile 3: social media-centric repertoire	Profile 4: comprehensive high-frequency repertoire	Profile 5: online-only repertoire
	***M*** **(SD)**	***M*** **(SD)**	***M*** **(SD)**	***M*** **(SD)**	***M*** **(SD)**
Financial journals	1.348 (0.049)	2.645 (0.207)	1.418 (0.072)	5.037 (0.173)	2.556 (0.256)
Financial newspapers	1.213 (0.030)	2.332 (0.197)	2.332 (0.050)	5.076 (0.204)	1.756 (0.119)
Financial broadcast & TV	1.820 (0.070)	3.517 (0.135)	2.439 (0.175)	5.474 (0.197)	4.873 (0.201)
Mobile financial info apps/sites	1.839 (0.073)	4.203 (0.158)	2.881 (0.270)	6.009 (0.178)	6.002 (0.153)
PC financial info sites	1.386 (0.044)	3.442 (0.184)	1.747 (0.240)	5.769 (0.216)	5.208 (0.264)
Forum-based financial communities	1.269 (0.036)	3.084 (0.168)	1.560 (0.153)	5.213 (0.234)	5.323 (0.255)
IM-based financial communities	1.833 (0.086)	3.993 (0.248)	4.621 (0.272)	5.946 (0.172)	6.097 (0.141)
Video-based financial influencers	2.157 (0.098)	4.338 (0.250)	5.730 (0.239)	5.975 (0.132)	6.222 (0.128)
Non-video-based financial influencers	1.664 (0.074)	3.856 (0.296)	5.971 (0.175)	5.871 (0.139)	6.064 (0.248)
Sample size (percentage)	386 (38.52%)	217 (21.66%)	152 (15.17%)	118 (11.78%)	129 (12.87%)

Usage frequency was measured on a 7-point scale: 1 = Never, 2 = 1–3 times/year, 3 = 1–3 times/half-year, 4 = 1–3 times/month, 5 = 1–3 times/week, 6 = 4–7 times/week, 7 = Multiple times daily.

Profile 1: Comprehensive Low-Frequency Repertoire (38.52%). This constitutes the largest group. Users in this profile exhibited consistently low usage frequencies across all nine media types, representing a group of “financial news avoiders.”

Profile 2: Comprehensive Medium-Frequency Repertoire (21.66%). This group showed moderate engagement across most platforms but maintained relatively lower usage of traditional print media (journals and newspapers).

Profile 3: Social Media-Centric Repertoire (15.17%). These users demonstrated distinctively high usage of social-driven platforms—specifically IM communities, video-based financial influencers, and non-video-based financial influencers—while engaging minimally with other channels.

Profile 4: Comprehensive High-Frequency Repertoire (11.78%). This segment represents the “omnivores” of financial information, reporting high frequency of use across both traditional and digital platforms.

Profile 5: Online-Only Repertoire (12.87%). This group is characterized by high engagement across all digital channels and broadcasting, while almost completely eschewing traditional print press (journals and newspapers).

### 4.3 Factors associated with financial media repertoire membership

To examine how socio-demographic traits, psychological motivations, and structural-environmental factors correlate with individuals' membership in distinct financial media repertoires, a multinomial logistic regression model was estimated. Given the cross-sectional design of this survey, all parameters reflect cross-sectional statistical associations rather than directional causal claims. The five-profile typology derived from Latent Profile Analysis (LPA) served as the categorical dependent variable, with Profile 1 (Comprehensive Low-Frequency Repertoire) designated as the baseline reference group. Full results are presented in Table 8.

**Table 8:** Multinomial logistic regression results (ref: profile 1 comprehensive low-frequency repertoire).

Predictors	Profile 2: medium-freq (*n* = 217)	Profile 3: social-centric (*n* = 152)	Profile 4: high-freq (*n* = 118)	Profile 5: online-only (*n* = 129)
	Exp (B)	Exp (B)	Exp (B)	Exp (B)
**15.6-7.4,14498ptDemographics**			
Age	0.998	0.973^**^	1.012	1.009
Gender (female)	0.831	1.097	0.656	0.638+
No systematic fin. learning	0.298^***^	0.750	0.206^***^	0.263^***^
15.6-7.4,-1498ptNon-finance occupation	0.924	3.794+	0.385^*^	0.790
**Motivations**	
Affective	1.012	1.290^*^	1.529^**^	1.145
Cognitive	1.715^***^	1.316^**^	1.871^***^	2.528^***^
Personal integration	1.014	0.876	0.998	0.911
Companionship	1.022	1.037	0.958	0.896
Belongingness	1.179	1.084	1.690^**^	1.458^*^
Escapism	0.912	0.756^*^	0.771+	0.750^*^
15.6-7.4,-1498ptHabitual use	1.073	1.170	0.936	1.100
Structural factors	
Work hours (level 2)	2.794	5.103	7.135+	1.937
Work hours (level 3)	2.396	4.686	7.049+	1.975
Commute time (level 1)	1.059	0.959	0.401^*^	2.212
Commute time (level 3)	0.980	0.881	0.255^*^	1.891
Region (non-municipality)	0.728	1.071	0.590	0.192^*^
Region (non-capital)	0.963	1.428	0.926	0.208+
Region (non-prefecture)	0.781	1.516	0.632	0.172^*^
Region (non-county city)	0.957	0.951	0.555	0.126^*^
McFadden *R*^2^	0.224			
−2 Log likelihood	2333.291			

The table presents only the core variables, with a large number of non-significant variables included in analysis but omitted for brevity. +*p* < 0.1, ^*^p < 0.05, ^**^p < 0.01, ^***^p < 0.001.

#### 4.3.1 Socio-demographic patterns and differences

Individual background characteristics, educational training, and occupational settings display significant associations with repertoire membership:

Lack of Systematic Financial Education: Compared to individuals with formal financial training, respondents lacking systematic financial learning showed significantly lower odds of belonging to active repertoires—specifically the Comprehensive Medium-Frequency Repertoire (Exp(B) = 0.298, *p* < 0.05), the Comprehensive High-Frequency Repertoire (Exp(B) = 0.206, *p* < 0.05), and the Online-Only Repertoire (Exp(B) = 0.263, *p* < 0.05)—by 70 to 80%. This indicates a strong positive association between formal financial education and engagement with high-density financial media.

Occupational Divergence: Relative to financial professionals, non-finance practitioners exhibited marginally higher odds of belonging to the Social Media-Centric Repertoire (Exp(B) = 3.794, *p* < 0.10), but lower odds of entering the Comprehensive High-Frequency Repertoire (Exp(B) = 0.385, *p* < 0.05). This contrast suggests that non-professionals tend to align more with socially embedded, lower-barrier channels, whereas professionals are more correlated with cross-platform omnivorous media usage.

Age and Gender Profiles: Older age was negatively associated with membership in the Social Media-Centric Repertoire (Exp(B) = 0.973, *p* < 0.01), pointing to a younger demographic composition in social-media-based financial information consumption. Additionally, female respondents showed marginally lower odds than male respondents of being in the Online-Only Repertoire (Exp(B) = 0.638, *p* < 0.10), revealing subtle gender differences in reliance on purely digital financial ecosystems.

#### 4.3.2 Psychological motivations and repertoire associations

Under the Uses and Gratifications perspective, specific psychological gratifications demonstrate distinct correlational patterns across different media profiles:

Universal Association of Cognitive Motivation: Cognitive motivation displayed a consistent, positive correlation with all active media groups relative to the baseline. Higher cognitive motivation predicted greater odds of belonging to the Comprehensive Medium-Frequency Repertoire (Exp(B) = 1.715, *p* < 0.05), the Social Media-Centric Repertoire (Exp(B) = 1.316, *p* < 0.05), the Comprehensive High-Frequency Repertoire (Exp(B) = 1.871, *p* < 0.05), and the Online-Only Repertoire (Exp(B) = 2.528, *p* < 0.05). The strongest association emerged for Profile 5 (Online-Only Repertoire), highlighting environmental surveillance as a key psychological correlate of digital media preference.

Selective Correlations of Affective and Belongingness Needs: Affective motivation was positively associated with membership in the Social Media-Centric Repertoire (Exp(B) = 1.290, *p* < 0.05) and the Comprehensive High-Frequency Repertoire (Exp(B) = 1.529, *p* < 0.05). Belongingness motivation uniquely correlated with higher odds of entering the Comprehensive High-Frequency Repertoire (Exp(B) = 1.690, *p* < 0.05) and the Online-Only Repertoire (Exp(B) = 1.458, *p* < 0.05), aligning emotional resonance and social identity with deeper, multi-channel media engagement.

Inverse Association of Escapism: Conversely, escapism motivation was negatively associated with active profile membership, including Profile 3 (Exp(B) = 0.756, *p* < 0.05), Profile 4 (Exp(B) = 0.771, *p*<*0.10*), and Profile 5 (Exp(B) = 0.750, *p* < 0.05). This indicates that individuals oriented toward stress avoidance are more likely to remain in the low-frequency group, as financial information carries high cognitive demands that may not serve escapist needs.

#### 4.3.3 Structural spatiotemporal and regional factors

Contextual lifestyle constraints and regional environments are strongly linked with media repertoire distribution:

Workplace Time vs. Transit Time: Relative to the longest working hours (Level 4), moderate work durations (Level 2 and 3) were marginally positively associated with belonging to the Comprehensive High-Frequency Repertoire (Exp(B) ≈ 7.0, *p* < 0.10). Conversely, shorter commute durations (Level 1 Exp(B) = 0.401, *p* < 0.05; Level 3 Exp(B) = 0.255, *p* < 0.05) were negatively associated with membership in the Comprehensive High-Frequency Repertoire compared to Level 4. This pattern indicates that omnivorous, high-frequency media engagement is more closely aligned with dedicated workplace time allocation than with fragmented transit consumption.

Metropolitan Agglomeration: For Profile 5 (Online-Only Repertoire), living in non-municipalities (Exp(B) = 0.192, *p* < 0.05), non-capital cities (Exp(B) = 0.208, *p* < 0.10), non-prefecture cities (Exp(B) = 0.172, *p* < 0.05), or non-county cities (Exp(B) = 0.126, *p* < 0.05) exhibited strong or marginally significant negative associations relative to their respective reference categories. This confirms that purely online financial media repertoires are significantly concentrated among metropolitan populations, reflecting regional alignment with digital infrastructures and financial ecosystems.

### 4.4 Incremental explanatory power: hierarchical regression analysis

To empirically evaluate whether psychological motivations offer unique incremental explanatory power beyond objective socio-structural constraints and pure instrumental rationality, a four-step hierarchical multinomial logistic regression analysis was conducted. Predictors were sequentially entered into four conceptual blocks. Repertoire membership (Profile 1: Comprehensive Low-Frequency Repertoire served as the reference category, *n* = 386) was specified as the dependent variable. Fit statistics and likelihood ratio tests across sequential models are summarized in Table 9.

**Table 9:** Hierarchical multinomial logistic regression model comparisons.

Model Step	Predictors entered in block	Model−2LL	Model χ^2^ (df)	McFadden R^2^	*Δχ* ^2^	Δdf	*p*-value of change
Model 1	Controls only (socio-demographics, work/commute, location)	2,584.121	421.883 (132)^***^	0.140	—	—	—
Model 2	+ instrumental rationality (**cognitive motivation**)	2,387.200	620.191 (136)^***^	0.206	196.921	4	< 0.001
Model 3	+ Social & identity integration (**personal, belongingness, companionship**)	2,358.574	648.816 (148)^***^	0.216	28.626	12	< 0.01
Model 4	+ Affective & psychological coping (**affective, escapism, habitual use**)	2,333.291	674.100 (160)^***^	0.224	25.283	12	< 0.05

Reference category is Profile 1 (*n* = 386). Total *N* = 1002. McFadden *R*^2^ increases sequentially from 0.140 to 0.224 (Nagelkerke *R*^2^ = 0.515 in Model 4). Δχ^2^ represents the likelihood ratio test statistic between sequential models. ^*^p < 0.05, ^**^p < 0.01, ^***^p < 0.001.

Model 1 established the baseline with socio-structural and functional controls (χ^2^ = 421.883, *df* = 132, *p* < 0.001; McFadden *R*^2^ = 0.140). The sequential entry of psychological blocks yielded statistically significant improvements in model fit at every step:

Model 2 (Δχ^2^ = 196.921, Δdf = 4, *p* < 0.001; McFadden *R*^2^ = 0.206) confirmed that instrumental cognitive surveillance forms the indispensable functional foundation for active media engagement (Exp(B)s = 1.316–2.528, *p* < 0.05 across profiles).

Model 3 (Δχ^2^ = 28.626, Δdf = 12, *p* = 0.004; McFadden *R*^2^ = 0.216) demonstrated that social belongingness provides unique explanatory power beyond simple utility, significantly elevating the odds of belonging to the Comprehensive High-Frequency Repertoire (Exp(B) = 1.541, *p* = 0.005) and the Online-Only Repertoire (Exp(B) = 1.386, *p* = 0.019).

Model 4 (Δχ^2^ = 25.283, Δdf = 12, *p* = 0.014; McFadden *R*^2^ = 0.224) revealed critical emotional and anxiety-avoidance mechanisms: Affective motivation strongly catalyzed membership in the Comprehensive High-Frequency Repertoire (Exp(B) = 1.529, *p* = 0.006), whereas escapism significantly reduced active repertoire adoption (e.g., Exp(B) = 0.750, *p* = 0.040 for Profile 5).

The highly significant sequential likelihood ratio changes (Δχ^2^(1 → 2): *p* < 0.001; Δχ^2^(2 → 3): *p* = 0.004; Δχ^2^(3 → 4): *p* = 0.014) provide decisive empirical evidence that financial media repertoire adoption is not a purely calculative, utilitarian choice. Instead, while cognitive surveillance forms the functional threshold, social belongingness, affective resonance, and psychological anxiety-avoidance constitute non-redundant, essential boundary conditions that drive audience repertoire differentiation.

### 4.5 Robustness checks and subsample heterogeneity

#### 4.5.1 Subsample distributional heterogeneity and robustness replications

Prior to evaluating model stability, a cross-tabulation analysis was conducted (Table 10) to examine baseline distributional heterogeneity across age (< = 35 vs. >35), geographic location (Core Urban vs. Non-Core Urban), and financial domain expertise (Insiders vs. Laypersons). As detailed in Table 10, Insiders (*n* = 297) exhibited a uniquely distinct repertoire pattern that stands in stark contrast to all other demographic and spatial cohorts. Specifically, Insiders recorded the lowest proportions in Profile 1 (Comprehensive Low-Frequency Repertoire, 16.84%) and Profile 3 (Social Media-Centric Repertoire, 7.07%)—whereas all other five subgroups ranged between 33.70–47.66% and 10.77–18.83%, respectively. Conversely, Insiders consistently outperformed every other subgroup in high-engagement and digital-native configurations, leading across Profile 2 (29.97%), Profile 4 (25.25%), and Profile 5 (20.88%). This baseline comparison confirms that financial domain expertise exerts a far more dominant pull than age or geography in driving active, multi-platform media repertoire selection.

**Table 10:** Demographic, spatial, and domain expertise subgroup distributions across financial media repertoires.

Subgroup stratification		Profile 1	Profile 2	Profile 3	Profile 4	Profile 5
Age stratification	≤ 35 Years (*n* = 547)	187 (34.19%)	122 (22.30%)	103 (18.83%)	63 (11.52%)	72 (13.16%)
	> 35 Years (*n* = 455)	199 (43.74%)	95 (20.88%)	49 (10.77%)	55 (12.09%)	57 (12.53%)
Geographic location	Core urban (*n* = 451)	152 (33.70%)	106 (23.50%)	61 (13.53%)	68 (15.08%)	64 (14.19%)
	Non-core urban (*n* = 551)	234 (42.47%)	111 (20.15%)	91 (16.52%)	50 (9.07%)	65 (11.80%)
Financial domain expertise	Insiders (*n* = 297)	50 (16.84%)	89 (29.97%)	21 (7.07%)	75 (25.25%)	62 (20.88%)
	Laypersons (*n* = 705)	336 (47.66%)	128 (18.16%)	131 (18.58%)	43 (6.10%)	67 (9.50%)

To rigorously test whether the primary empirical findings remain structurally stable against these sampling and baseline variations, the full multinomial logistic regression model (Model 4) was re-estimated across these split subsamples. As detailed in Table 11, the core motivational structure demonstrated remarkable consistency across subsamples despite the distributional disparities identified in Table 10. Cognitive motivation maintained a universally robust, positive prediction for active repertoire adoption. Similarly, Social Integration-Belongingness consistently propelled active media consumption in key subgroups. This stable replication confirms that the primary conclusions are not driven by sample aggregation artifacts.

**Table 11:** Subsample heterogeneity analysis: odds ratios of motivations predicting active financial media repertoires.

Predictors	Age stratification		Geographic Location		Financial Domain Expertise	
	≤ **35 years (*****n*** = **547)**	> **35 years (*****n*** = **455)**	**Core urban (*****n*** = **451)**	**Non-core urban (*****n*** = **551)**	**Insiders (*****n*** = **297)**	**Laypersons (*****n*** = **705)**
**15.6-7.4,14498ptProfile 2: comprehensive medium-frequency repertoire**			
Affective	0.957	1.032	0.793	1.104	0.946	1.106
Cognitive	**1.750** ^**^	**1.891** ^***^	**2.164** ^***^	**1.740** ^***^	**1.532+**	**1.805** ^***^
Personal integration	1.165	0.939	**0.672** ^*^	1.238	0.643	1.188
Companionship	0.909	1.067	1.155	0.934	0.921	1.006
Belongingness	1.252	1.205	**1.932** ^**^	0.837	1.783	1.022
Escapism	0.848	1.126	**0.687+**	1.220	0.740	0.979
15.6-7.4,-1498ptHabitual use	**1.313+**	0.802	1.122	1.070	1.490	0.968
**Profile 3: social media-centric repertoire**			
Affective	1.094	**1.786** ^**^	1.094	**1.388** ^*^	1.188	**1.292** ^*^
Cognitive	**1.441** ^*^	**1.445** ^*^	**1.661** ^**^	1.227	**1.744+**	**1.273** ^*^
Personal integration	0.874	0.713	**0.600** ^*^	1.048	0.863	0.888
Companionship	1.091	1.138	1.008	1.084	0.428	1.182
Belongingness	0.982	1.273	1.506	0.898	0.810	1.013
Escapism	0.767	0.730	**0.658+**	0.813	1.231	**0.724** ^*^
15.6-7.4,-1498ptHabitual use	**1.333+**	0.932	1.291	1.140	1.592	1.213
**Profile 4: comprehensive high-frequency repertoire**			
Affective	**1.906** ^*^	**1.734** ^*^	1.479	**1.550+**	1.570	**1.524+**
Cognitive	1.285	**2.294** ^**^	**2.123** ^*^	**1.881** ^*^	**1.920+**	**1.766** ^*^
Personal integration	1.377	0.830	0.689	1.160	0.964	1.161
Companionship	**0.602+**	1.113	0.857	0.887	0.620	1.293
Belongingness	**1.700** ^*^	**1.752** ^*^	**3.002** ^***^	1.292	**3.149** ^**^	1.250
Escapism	0.838	0.678	**0.552** ^*^	0.988	**0.546+**	0.720
15.6-7.4,-1498ptHabitual use	1.083	0.951	1.021	1.031	1.273	0.938
**Profile 5: online-only repertoire**			
Affective	1.030	1.248	0.996	1.145	1.107	1.197
Cognitive	**2.840** ^***^	**2.858** ^***^	**3.248** ^***^	**2.546** ^***^	**2.791** ^**^	**2.739** ^***^
Personal integration	0.983	0.723	**0.651+**	1.110	0.742	0.972
Companionship	0.767	1.055	0.988	0.813	0.847	0.838
Belongingness	**1.732** ^*^	1.419	**2.203** ^**^	1.110	**2.084+**	**1.361+**
Escapism	0.829	**0.660+**	**0.614** ^*^	0.795	0.594	0.762
Habitual use	1.064	1.159	1.092	1.271	1.181	1.134
McFadden *R*^2^	0.303	0.274	0.291	0.261	0.293	0.212

Reference category is Profile 1 (*n* = 386). Values are Odds Ratios [OR = Exp(B)] predicting profile membership relative to baseline. All subsample models achieved overall statistical significance (*p* < 0.001). Bolded values indicate statistical or marginal significance. + *p* < 0.10, ^*^ *p* < 0.05, ^**^ *p* < 0.01, ^***^ *p* < 0.001.

#### 4.5.2 Heterogeneity analyses and boundary conditions

Cross-group comparisons across the three stratifications delineate critical boundary conditions under which specific psychological drivers are amplified, attenuated, or structurally shifted.

(1) Generational Dynamics (Age Stratification)

Cognitive Universalism vs. High-Frequency Shift: Cognitive surveillance operates as a universal driver for Profile 5 (Online-Only Repertoire) across both younger (Exp(B) = 2.840, *p* < 0.001) and older audiences (Exp(B) = 2.858, *p* < 0.001). However, for Profile 4 (Comprehensive High-Frequency Repertoire), Cognitive motivation is only statistically significant among older adults (Exp(B) = 2.294, *p* < 0.01), whereas it is non-significant for youth (Exp(B) = 1.285, *p* > 0.1).

Belongingness and Affective Divergence: for younger digital natives, Belongingness is a vital catalyst for switching to the Online-Only Repertoire (Exp(B) = 1.732, *p* < 0.05), a pattern that fades among older adults (Exp(B) = 1.419, *p* > 0.1). Conversely, older audiences seek emotional resonance primarily through the Social Media-Centric Repertoire (Exp(B) = 1.786, *p* < 0.01), whereas younger adults rely on Affective motivation to maintain high-frequency multi-channel consumption (Exp(B) = 1.906, *p* < 0.05). Additionally, younger users exhibit a marginally significant, habit-driven inertia toward light media consumption (Profile 2 Habitual Use: Exp(B) = 1.313, *p*<*0.10*; Profile 3 Habitual Use: Exp(B) = 1.333, *p* < 0.10).

(2) Spatial Contextualization (Geographic Stratification)

Urban Belongingness Exclusivity: social Integration-Belongingness exhibits pronounced urban exclusivity. In Core Urban environments, Belongingness acts as a dominant engine, strongly propelling membership in Profile 2 (Exp(B) = 1.932, *p* < 0.01), Profile 4 (Exp(B) = 3.002, *p* < 0.001), and Profile 5 (Exp(B) = 2.203, *p* < 0.01). In sharp contrast, Belongingness exerts zero statistically significant impact across all profiles in Non-Core Urban areas (*p* > 0.1).

Metropolitan Stress and Anxiety Avoidance: core urban residents display a consistent anxiety-avoidance mechanism. Escapism motivation significantly or marginally suppresses financial news consumption across virtually all active repertoires (Profile 2: Exp(B) = 0.687, *p*<*0.10*; Profile 3: Exp(B) = 0.658, *p*<*0.10*; Profile 4: Exp(B) = 0.552, *p* < 0.05; Profile 5: Exp(B) = 0.614, *p* < 0.05). In high-density urban settings, financial news serves as mandatory social currency, but its high cognitive load causes stress-seeking individuals to actively withdraw. Additionally, Personal Integration shows a negative suppression in Core Urban settings (Profile 2: Exp(B) = 0.672, *p* < 0.05; Profile 3: Exp(B) = 0.600, *p* < 0.05).

(3) Capital-Driven Mechanisms (Financial Domain Expertise)

Domain Insiders (Pragmatic Professionalism and Identity): for Insiders, media choice is driven by strict utility and professional identity. Cognitive motivation (Exp(B) = 2.791, *p* < 0.01) and Belongingness (Exp(B) = 3.149, *p* < 0.01; Profile 5: Exp(B) = 2.084, *p* < 0.10) dominate their engagement with the Online-Only Repertoire and the Comprehensive High-Frequency Repertoire. Crucially, Affective motivation is completely non-significant across all profiles (*p* > 0.1), confirming that domain experts approach financial media with emotional detachment.

Domain Laypersons (Affective and Cognitive Compensation): For Laypersons, lacking domain expertise reactivates Affective motivation, which significantly catalyzes engagement with the Social Media-Centric Repertoire (Profile 3: Exp(B) = 1.292, *p* < 0.05) and marginally significantly catalyzes engagement with the Comprehensive High-Frequency Repertoire (Profile 4: Exp(B) = 1.524, *p* < 0.10). Lay audiences rely heavily on affective resonance and engaging presentation to cushion the dense cognitive threshold of financial news.

## 5 Discussion

This study aimed to deconstruct the heterogeneity of financial information consumption among Chinese users by identifying distinct media repertoires and examining their psychological and structural determinants. By applying a person-centered approach, this study identified five distinct media repertoire typologies. The results reveal that financial information acquisition is not a monolithic rational process but a stratified behavioral outcome shaped by the interplay of financial domain expertise (encompassing educational and professional background), affective vs. cognitive motivations, and structural availability.

### 5.1 Financial media repertoire typologies in the digital age

By evaluating financial media consumption through a person-centered, multi-platform lens (RQ1), this study identifies five distinct user typologies: Comprehensive Low-Frequency Repertoire (Profile 1, 38.5%), Comprehensive Medium-Frequency Repertoire (Profile 2, 21.7%), Social Media-Centric Repertoire (Profile 3, 15.2%), Comprehensive High-Frequency Repertoire (Profile 4, 11.8%), and Online-Only Repertoire (Profile 5, 12.9%). The empirical results reveal an overwhelming shift toward online platforms across all profiles. This universal digital reliance empirically confirms the profound reconfiguration of financial news dissemination. Yet, this digital migration does not yield a uniform behavioral practice, but rather a highly stratified ecosystem of multi-platform engagement.

A prominent finding of this study is the pervasive digital migration and the absolute dominance of video-based financial influencers, which emerged as the primary information source across virtually all user profiles—ranging from low-frequency consumers to online seekers. This visual and algorithmic turn reinforces Wang's (2025) observation that short-video feeds have reconstructed the infrastructure of financial knowledge dissemination in China. By translating complex economic metrics into narrative storytelling and visual graphics, video influencers provide “multimodal cognitive de-friction,” drastically lowering the operational and decoding barriers for financial media users.

Crucially, among active digital consumers, the analysis reveals that audience repertoire configurations bifurcate into two structural patterns:

Pattern 1: Open, Cross-Platform Integration: Typified by the Online-Only Repertoire (Profile 5) and the Comprehensive High-Frequency Repertoire (Profile 4), users actively synthesize specialized financial APPs, digital communities, and video feeds into a synergistic, multi-layered information diet.

Pattern 2: Closed, Algorithmic Ecosystem Confinement: Typified by the Social Media-Centric Repertoire (Profile 3), users confine their entire media diet strictly to a single category of media—namely, socially driven and algorithmically curated channels (e.g., instant messaging communities, short-video feeds, and social influencers)—demonstrating a highly dependent, category-bound engagement pattern.

This structural divergence extends Hargittai's (2002) “second-level digital divide” into the domain of specialized financial media. Expanded digital access does not automatically lead to comprehensive, cross-platform financial information consumption; rather, it induces structural differentiation. A substantial proportion of users adopt a social media-centric financial media usage pattern, and this reliance on social media for financial information further fosters an illusion of blind overconfidence—specifically, where audiences display elevated subjective financial literacy despite deficient objective literacy (Olajide et al., 2024). This, in turn, drives uninformed financial decision-making, leaving users exposed to heightened market volatility and misinformation (Shu et al., 2020; Hu and Liu, 2025).

### 5.2 Motivational drivers and contextual boundary conditions of repertoire configurations

#### 5.2.1 Multi-dimensional motivational architecture: beyond pure instrumental rationality

The empirical analysis of this study reveals that audience navigation across cross-platform financial media is governed by a multi-dimensional motivational architecture. Instrumental rationality, represented by cognitive motivation, constitutes the primary baseline driver of financial media consumption. As shown in the model progression, introducing cognitive motivations into the baseline model yields the most substantial single-block improvement in overall explanatory power. This trend aligns with established media exposure scholarship (Knobloch-Westerwick et al., 2019), confirming that functional information surveillance and problem-solving utility remain indispensable core drivers prompting audiences to initiate financial media engagement.

However, a purely rational or cognition-centric perspective leaves critical behavioral variation unexplained. The sequential addition of social-identity integration and psychological coping motivators generates statistically significant incremental gains in model fit. This finding extends recent scholarship in digital financial communication (Ahern and Peress, 2023; Ferah and Sümer, 2023) by demonstrating that digital financial media choices are not dictated solely by objective information retrieval needs. In an increasingly complex and volatile digital financial environment, audiences actively utilize financial media as a mechanism for psychological adaptation, where affective motivations and social drivers (e.g., motivation of belongingness) provide unique and indispensable incremental explanatory power beyond functional utility. This aligns with Mood Management Theory (Zillmann, 1988) and self-concept management models (Knobloch-Westerwick, 2015), which posit that media selection serves not only to retrieve information but also to optimize affective states and cultivate a sense of belonging.

Notably, the psychological adaptation capacity of financial media must be strictly distinguished from generic escapism—namely, using media as a vehicle to procrastinate or withdraw from daily obligations such as work, school, or family. The empirical findings demonstrate that escapism consistently acts as a significant negative predictor across all active financial media repertoires. This inverse relationship highlights the distinct cognitive demands of financial media: unlike leisure entertainment, which readily serves as a low-effort outlet for task avoidance or interpersonal detachment, financial media engagement requires purposeful cognitive involvement. Consequently, individuals seeking to postpone responsibilities or flee routine burdens actively avoid financial media repertoires, echoing broader patterns of intentional news avoidance driven by information fatigue and stress-coping strategies (Villi et al., 2022). This further reinforces that financial media consumption—even when serving affective coping functions—remains fundamentally effortful and goal-directed.

Taken together, the findings reveal a clear functional division of labor across motivational dimensions: cognitive motivation is closely associated with moderate-to-high frequency repertoire choices, serving as the baseline gateway for audiences to transcend media avoidance or low-frequency use. Conversely, socio-affective and psychological coping motivations determine the structural configuration of the repertoire itself—dictating whether users engage in open, cross-platform exploration or retreat into enclosed, social-centric financial media ecosystems. Ultimately, financial media consumption emerges as a continuous, dynamic process of balancing rational information utility with affective psychological adaptation.

#### 5.2.2 Structural constraints and affordances: spatiotemporal routines and regional infrastructure

Beyond individual psychological agency, the empirical findings forcefully reaffirm Webster's (2011) Duality of Media framework by demonstrating that cross-platform financial media repertoires are fundamentally bounded and enabled by structural spatiotemporal conditions.

First, in terms of temporal structure, the empirical results demonstrate that membership in the Comprehensive High-Frequency Repertoire (Profile 4) is tightly bound to audience time availability, structured by both workplace and commute hours. This pattern underscores the critical role of structural availability in shaping domain-specific information diets (Taneja et al., 2012; Yuan and Webster, 2006). On one hand, moderate workplace durations (Levels 2 and 3) provide the necessary daily rhythms and institutional affordances to sustain continuous, multi-channel financial surveillance. On the other hand, commute duration functions through a balanced temporal equilibrium: with the longest commute tier (Level 4) serving as the reference benchmark, both minimal (Level 1) and intermediate (Level 3) commutes significantly depress the likelihood of adopting the Comprehensive High-Frequency Repertoire, whereas moderate commutes (Level 2) exhibit statistical equivalence to Level 4. Together, these findings reveal that maintaining an omnivorous financial news diet requires stable, low-friction time windows: while minimal commutes lack sufficient exposure duration and intermediate commutes suffer from transit fragmentation (plausibly burdened by higher-friction transfers or driving), extended transit windows (Level 4) likely afford dedicated, uninterrupted blocks of mobile screen time necessary for immersive, multi-channel news engagement.

Second, in terms of spatial structure, regional urban hierarchy acts as a structural catalyst for digital media adoption. The empirical findings demonstrate that across all urban administrative tiers—ranging from municipalities and capital cities to prefecture and county-level cities—the odds of belonging to the Online-Only Repertoire (Profile 5) are substantially higher compared to rural contexts. This pattern confirms that the curation of an exclusively digital-native financial media diet is broadly concentrated across urbanized areas (i.e., regions outside rural villages). In urban centers of all administrative ranks, denser informational environments, higher localized economic activity, and ambient financial engagement systematically lower the friction of adopting specialized online platforms (Kim, 2016).

#### 5.2.3 Contextual boundaries and audience heterogeneity: identity anchoring, generational divergence, and psychological buffering

The subsample heterogeneity analysis delineates key contextual boundary conditions governing financial media repertoire choices. The empirical findings yield three critical patterns, further enriching contemporary communication scholarship and audience repertoire frameworks.

First, the predictive role of belongingness motivation across active media repertoires exhibits pronounced geographic and professional divergence. The strong driving effect of belongingness on the Comprehensive High-Frequency Repertoire (Profile 4) and the Online-Only Repertoire (Profile 5) is concentrated predominantly among core urban residents and financial domain insiders. In high-density metropolitan areas and specialized professional circles, cross-platform financial media engagement transcends mere information retrieval, evolving into an identity-anchoring mechanism that reinforces self-identity and social connection (e.g., Swart et al., 2017). Conversely, for non-core urban and non-expert populations, financial media retains a strictly functional utility, lacking the capacity for social identity construction.

Second, a distinct generational split characterizes the motivational architecture of the Comprehensive High-Frequency Repertoire adoption (Profile 4). For younger audiences ( ≤ 35 years), high-frequency cross-platform engagement is driven primarily by affective and belongingness motivations, while cognitive motivation ceases to be statistically significant. For older, mature audiences (>35 years), however, high-frequency use remains firmly driven by dual cognitive and affective motivators. This empirical finding echoes Anderson's (2025) study on generational cohort differences in news consumption, indicating that mature audiences' financial media use remains tightly linked to cognitive needs. Conversely, younger digital natives tend to navigate multi-platform environments primarily as a socio-emotional habitat. Furthermore, Swart (2023) reveals that young adults' cross-platform interaction serves mainly to foster peer connection, negotiate digital identity, and regulate emotional states. As Notley et al. (2023) emphasize, youth news engagement is inherently relational and affective rather than an isolated pursuit of knowledge. Consequently, high-frequency financial media engagement in this context may function predominantly as a vehicle for youth sociability.

Third, affective motivation operates as an indispensable “psychological buffer” for non-expert and non-central sub-groups within the Social Media-Centric Repertoire (Profile 3). Affective motivation significantly increases the odds of Profile 3 adoption among older adults, non-core urban residents, and laypersons, whereas it's predictive effect is non-significant for younger cohorts or core urban elites. This divergence reflects an alignment between audience cognitive characteristics and mobile social media exposure patterns: on the one hand, older adults, non-core urban residents, and less-educated populations in China face a pronounced gap in digital literacy and information-processing capacity (Wang et al., 2023). On the other hand, mobile-based social media news exposure is predominantly characterized by “news snacking”—a fragmentary reading pattern requiring low attention investment (Molyneux, 2018). Consequently, audience groups with relatively constrained financial and digital literacy lean toward social-centric media ecosystems to consume information at low cognitive costs during routine leisure, bypassing the heavy processing demands of specialized financial journalism.

### 5.3 Theoretical contributions and targeted practical implications

#### 5.3.1 Theoretical contributions

This study makes three incremental theoretical contributions to financial communication scholarship and audience repertoire frameworks.

First, it expands Uses and Gratifications (U&G) theory from an isolated single-medium perspective into a repertoire ecology framework. Traditional U&G paradigms predominantly focus on audiences' isolated choices regarding single media outlets (Katz et al., 1973). Overcoming this limitation, this study demonstrates how a multidimensional motivational architecture drives audiences to configure structured, cross-platform media repertoires, thereby responding to and refining audience repertoire theory in digital communication environments (Yuan, 2011; Swart et al., 2017).

Second, it reevaluates affective motives in specialized media selection by uncovering an “affective buffering effect” under cognitive constraints. Prior scholarship has largely emphasized the functional role of media in delivering financial information (Ahern and Peress, 2023; Ferah and Sümer, 2023). The empirical findings of this study directly engage with the “emotional turn” in news scholarship (Wahl-Jorgensen, 2020), illustrating that digital news consumption dissolves the binary opposition between “rationality and emotionality,” as audiences lean toward more relatable and resonant expressions. Affective motivation exhibits a distinct predictive power in driving lay audiences toward the Social Media-Centric Repertoire, revealing that affective motives function as a psychological buffer connecting non-expert audiences to the financial information ecosystem during low-cognitive-load “news snacking” exposure (Molyneux, 2018).

Third, it delineates spatial and generational boundary conditions for repertoire theory. The predictive effects of motivations on media repertoire selection are not universal, but rather depend heavily on the social context in which audiences are embedded. This study clarifies how geographic space (core vs. non-core urban areas) and generational cohorts (younger vs. mature audiences) act as critical boundary conditions that moderate the predictive pathways of psychological motivations on repertoire choices: among core urban residents and domain insiders, belongingness motivation—embodying an identity-anchoring mechanism (Swart et al., 2017)—drives the adoption of high-frequency cross-platform repertoires; conversely, among young digital natives, affective and belongingness motives—reflecting a socio-emotional habitat construction pathway (Notley et al., 2023; Anderson, 2025)—drive the configuration of multi-platform repertoires.

#### 5.3.2 Targeted practical and behavioral interventions

First, for professional financial media organizations and content creators, strategic emphasis must shift toward video-first and multimodal content distribution. Empirical results indicate that audiences have established an online-centric—and specifically online video-centric—financial information consumption pattern. To maximize engagement, traditional and professional creators should pivot from text-heavy reporting toward short-form visual explainers, data visualization, and video-based analytical coverage, meeting audience preferences for visually intuitive and real-time news formats.

Second, for digital platforms, feature designs and algorithmic architectures should actively reinforce socio-emotional connection and community interaction. The findings reveal that financial media platforms function not merely as information conduits, but as vital “socio-emotional habitats” accommodating users' intrinsic motivations for emotional relief, companionship, and belongingness. Platforms should move beyond basic content delivery to embed peer-interaction spaces, empathetic discussion nodes, and community-building attributes, cultivating a supportive network environment that satisfies users' relational and emotional needs.

Third, for financial regulators and literacy educators, policy interventions must prioritize targeted national financial literacy initiatives to mitigate social-media-induced behavioral biases. Findings demonstrate that various social media platforms serve as vital conduits for financial information, with a distinct subset of users adopting the Social Media-Centric Repertoire. Unanchored opinions and peer interactions on social media frequently exacerbate individual behavioral biases, creating “echo chamber” effects (Cookson et al., 2023) that prove particularly detrimental to users within the Social Media-Centric Repertoire. By contrast, financially literate Insiders rarely adopt this repertoire, nor do their motivations propel its formation. In light of this, regulators should deploy tailored literacy campaigns specifically targeting non-core urban residents and laypersons to buffer against peer distortion and subjective market noise.

### 5.4 Limitations and future research directions

Several limitations of this study should be acknowledged, which open up valuable avenues for future inquiry:

First, regarding sampling and generalizability, although the sample size (*N* = 1,002) ensures sufficient statistical power for Latent Profile Analysis, reliance on online quota sampling may underrepresent cohorts with lower digital or financial literacy, constraining broad population-level generalizability. Second, regarding measurement granularity, the survey categorized broad structural media channels (e.g., social media, online video platforms) rather than capturing platform-specific algorithmic recommendation feeds or granular platform metrics (e.g., Douyin, Xiaohongshu, Bilibili). Third, regarding research design and causal inference, the cross-sectional design precludes strict causal conclusions and remains susceptible to potential self-report recall bias and endogeneity concerns.

To address these limitations, future research should pursue two strategic directions:

First, scholars should deploy longitudinal panel tracking, randomized scenario experiments (between-subjects designs), or identify exogenous instrumental variables (IVs) to isolate directional causal mechanics between psychological motivations, repertoire transitions, and downstream financial behaviors.

Second, future studies should combine self-reported survey measures with objective digital trace data—such as platform API logs, app usage metering, or screen-time tracking—to unpack how algorithmic architectures dynamically shape information exposure.

## 6 Conclusion

By mapping how Chinese audiences navigate multi-platform financial information, this study demonstrates that digital migration does not homogenize media diets, but rather stratifies them into open, cross-platform integration versus enclosed, social media-centric consumption. Grounded in Webster's (2011) Duality of Media framework, this structural bifurcation reflects a dynamic where individual psychological agency—encompassing cognitive surveillance, identity anchoring, and affective buffering—is recursively bounded by daily spatiotemporal routines and regional urban hierarchies. Building on these empirical insights, this study advances targeted practical implications for three key stakeholders: professional financial media, digital platforms, and policy regulators. By tailoring multimodal content strategies, fostering supportive socio-emotional spaces, and deploying targeted literacy interventions, these stakeholders can collectively build a more inclusive and balanced financial communication ecosystem.

## Data Availability

The raw data supporting the conclusions of this article will be made available by the authors, without undue reservation.
