# Supplementary material for: Beyond information seeking: a latent profile analysis of financial media repertoires and psychological motivations among Chinese users
Source: Front Psychol. 2026 Sep 3;17:1757025. doi: 10.3389/fpsyg.2026.1757025 (PMC13582471; doi:10.3389/fpsyg.2026.1757025)
Supplement: Supplementary file 1 [file Table_1.docx]

Supplementary Material

# 1 Supplementary Figures and Tables

## 1.1 Supplementary Tables

Table S1. Original Results of Multinomial Logistic Regression Analysis Predicting Financial Media Repertoire Membership(Reference Group: Comprehensive Low-Frequency Users)

| Predictors | **Profile 2: Comprehensive Medium-Frequency** | | **Profile 3: Social Media-Centric** | | **Profile 4: Comprehensive High-Frequency** | | **Profile 5: Online-Only Users** | |
| --- | --- | --- | --- | --- | --- | --- | --- | --- |
|  | B | Exp(B) | B | Exp(B) | B | Exp(B) | B | Exp(B) |
| Intercept | -23.538 |  | -36.103 |  | -5.914* |  | -2.156 |  |
| Age | -0.002 | 0.998 | -0.028** | 0.973 | 0.011 | 1.012 | 0.009 | 1.009 |
| Motivation: Affective | 0.012 | 1.012 | 0.254* | 1.290 | 0.425** | 1.529 | 0.135 | 1.145 |
| Motivation: Cognitive | 0.540*** | 1.715 | 0.274** | 1.316 | 0.627*** | 1.871 | 0.927*** | 2.528 |
| Motivation: Personal Integration | 0.013 | 1.014 | -0.133 | 0.876 | -0.002 | 0.998 | -0.093 | 0.911 |
| Motivation: Companionship | 0.022 | 1.022 | 0.036 | 1.037 | -0.043 | 0.958 | -0.110 | 0.896 |
| Motivation: Belongingness | 0.164 | 1.179 | 0.081 | 1.084 | 0.525** | 1.690 | 0.377* | 1.458 |
| Motivation: Escape Reality | -0.092 | 0.912 | -0.279* | 0.756 | -0.260+ | 0.771 | -0.288* | 0.750 |
| Motivation: Habit | 0.070 | 1.073 | 0.157 | 1.170 | -0.066 | 0.936 | 0.096 | 1.100 |
| Gender(Ref: Male) | | | | | | | | |
| Female | -0.185 | 0.831 | 0.093 | 1.097 | -0.422 | 0.656 | -0.45+ | 0.638 |
| Systematic Fin. Learning (Ref: Yes) | | | | | | | | |
| No | -1.210*** | 0.298 | -0.287 | 0.750 | -1.581*** | 0.206 | -1.335*** | 0.263 |
| Finance-related Occupation (Ref: Yes) | | | | | | | | |
| No | -0.079 | 0.924 | 1.333+ | 3.794 | -0.955* | 0.385 | -0.236 | 0.790 |
| Region (Ref: Rural Area) | | | | | | | | |
| Non-Municipality | -0.317 | 0.728 | 0.069 | 1.071 | -0.528 | 0.590 | -1.650* | 0.192 |
| Non-Provincial Capital | -0.038 | 0.963 | 0.356 | 1.428 | -0.077 | 0.926 | -1.572+ | 0.208 |
| Non-Prefecture-level City | -0.247 | 0.781 | 0.416 | 1.516 | -0.460 | 0.632 | -1.758* | 0.172 |
| Non-County-level City | -0.044 | 0.957 | -0.050 | 0.951 | -0.588 | 0.555 | -2.071* | 0.126 |
| Personal Income (Ref: Level 7 - Highest) | | | | | | | | |
| Level 1 | 20.056 | 5.132E+08 | 14.521 | 2.025E+06 | 0.378 | 1.460 | 0.611 | 1.843 |
| Level 2 | 20.445 | 7.569E+08 | 14.240 | 1.529E+06 | 0.666 | 1.947 | 1.202 | 3.327 |
| Level 3 | 20.297 | 6.526E+08 | 14.144 | 1.389E+06 | 0.465 | 1.592 | 0.979 | 2.662 |
| Level 4 | 20.946 | 1.249E+09 | 14.675 | 2.363E+06 | 0.766 | 2.150 | 1.304 | 3.683 |
| Level 5 | 19.711 | 3.635E+08 | 13.098 | 4.882E+05 | 0.726 | 2.067 | 1.254 | 3.504 |
| Level 6 | 39.332 | 1.207E+17 | 15.956 | 8.502E+06 | 19.805 | 3.991E+08 | 19.054 | 1.883E+08 |
| Household Income (Ref: Level 7 - Highest) | | | | | | | | |
| Level 1 | -0.142 | 0.868 | 16.621 | 1.654E+07 | -0.500 | 0.606 | -1.491 | 0.225 |
| Level 2 | -0.498 | 0.607 | 17.051 | 2.541E+07 | -0.901 | 0.406 | -0.303 | 0.738 |
| Level 3 | -0.275 | 0.760 | 16.904 | 2.195E+07 | 0.055 | 1.057 | -0.358 | 0.699 |
| Level 4 | -0.061 | 0.940 | 17.169 | 2.861E+07 | 0.009 | 1.009 | 0.035 | 1.035 |
| Level 5 | -0.503 | 0.605 | 17.399 | 3.599E+07 | 0.562 | 1.754 | 0.430 | 1.538 |
| Level 6 | -0.068 | 0.934 | 16.563 | 1.560E+07 | -0.396 | 0.673 | 0.131 | 1.140 |
| Education Level (Ref: Level 5 - Highest) | | | | | | | | |
| Level 1 | -0.786 | 0.455 | 0.087 | 1.091 | -0.664 | 0.515 | -1.067 | 0.344 |
| Level 2 | 0.109 | 1.115 | -0.170 | 0.843 | -0.522 | 0.594 | -0.127 | 0.881 |
| Level 3 | -0.280 | 0.756 | -0.176 | 0.839 | -0.202 | 0.817 | 0.036 | 1.037 |
| Level 4 | 0.237 | 1.267 | -0.223 | 0.800 | 0.411 | 1.508 | 0.175 | 1.192 |
| Time at Home (Ref: Level 4 - Longest) | | | | | | | | |
| Level 1 | 0.312 | 1.367 | -0.151 | 0.860 | -0.002 | 0.998 | 0.056 | 1.057 |
| Level 2 | 0.560 | 1.751 | 0.071 | 1.073 | 0.330 | 1.392 | 0.080 | 1.083 |
| Level 3 | 0.600 | 1.821 | -0.233 | 0.792 | 0.207 | 1.230 | 0.129 | 1.138 |
| Work Hours (Ref: Level 4 - Longest) | | | | | | | | |
| Level 1 | 0.910 | 2.484 | 1.562 | 4.768 | 1.310 | 3.705 | 0.491 | 1.633 |
| Level 2 | 1.027 | 2.794 | 1.630 | 5.103 | 1.965+ | 7.135 | 0.661 | 1.937 |
| Level 3 | 0.874 | 2.396 | 1.545 | 4.686 | 1.953+ | 7.049 | 0.681 | 1.975 |
| Commute Time(Ref: Level 4 - Longest) | | | | | | | | |
| Level 1 | 0.057 | 1.059 | -0.042 | 0.959 | -0.913* | 0.401 | 0.794 | 2.212 |
| Level 2 | -0.002 | 0.998 | -0.064 | 0.938 | -0.156 | 0.855 | 0.587 | 1.798 |
| Level 3 | -0.02 | 0.980 | -0.127 | 0.881 | -1.368* | 0.255 | 0.637 | 1.891 |
| McFadden R^2^ | 0.224 | | | | | | | |
| -2 Log Likelihood | 2333.291 | | | | | | | |

Note: + p < 0.1, * p < 0.05, ** p < 0.01, *** p < 0.001;
